# Animal Approaches to Studying Risk Factors for Parkinson’s Disease: A Narrative Review

**DOI:** 10.3390/brainsci14020156

**Published:** 2024-02-02

**Authors:** R. H. Silva, L. B. Lopes-Silva, D. G. Cunha, M. Becegato, A. M. Ribeiro, J. R. Santos

**Affiliations:** 1 Behavioral Neuroscience Laboratory, Department of Pharmacology, Universidade Federal de São Paulo, São Paulo 04021-001, SP, Brazil; leonardobrito.biomed@gmail.com (L.B.L.-S.); deboragomesbio@gmail.com (D.G.C.); marcela.becegato@unifesp.br (M.B.); 2 Laboratory of Neuroscience and Bioprospecting of Natural Products, Department of Biosciences, Universidade Federal de São Paulo, Santos 11015-020, SP, Brazil; alessandra.ribeiro@unifesp.br; 3 Behavioral and Evolutionary Neurobiology Laboratory, Department of Biosciences, Federal University of Sergipe, Itabaiana 49500-000, SE, Brazil; joseronaldosantos@academico.ufs.br

**Keywords:** animal models, neurodegenerative disorder, prodromal signs, risk factors

## Abstract

Despite recent efforts to search for biomarkers for the pre-symptomatic diagnosis of Parkinson’s disease (PD), the presence of risk factors, prodromal signs, and family history still support the classification of individuals at risk for this disease. Human epidemiological studies are useful in this search but fail to provide causality. The study of well-known risk factors for PD in animal models can help elucidate mechanisms related to the disease’s etiology and contribute to future prevention or treatment approaches. This narrative review aims to discuss animal studies that investigated four of the main risk factors and/or prodromal signs related to PD: advanced age, male sex, sleep alterations, and depression. Different databases were used to search the studies, which were included based on their relevance to the topic. Although still in a reduced number, such studies are of great relevance in the search for evidence that leads to a possible early diagnosis and improvements in methods of prevention and treatment.

## 1. Introduction

Animal models are important resources for studying human diseases as they allow for the investigation of pathophysiological mechanisms and the screening of potential treatments. There are several protocols used in experimental studies of PD, both in non-human animals (mainly rodents) and cell culture. As illustrated in Table 1, the main animal models used for this purpose encompass pharmacological and genetic approaches [1,2,3,4,5,6,7]. In general, they achieve the validities that certify animal models [6,7], based on the core characteristics of the disease: hypofunction of the dopaminergic nigrostriatal pathway (construct validity) and the presence of motor alterations (face validity), which are ameliorated by classical antiparkinsonian drugs such as L-DOPA (predictive validity). Important additions to the construct aspects of many of these protocols are oxidative stress-induced damage, neuroinflammation, and increased levels of alpha-synuclein—although Lewy bodies (classical pathological hallmarks) are difficult to observe in PD animal models. Regarding phenomenological validity, several aspects of the deterioration of motor function are presented in similar or equivalent ways in relation to humans, such as akinesia, bradykinesia, rigidity, tremor, and balance alterations. Nevertheless, the gradual appearance of these alterations is not always achieved due to acute severe neuronal injuries caused by most protocols. In addition, non-motor symptoms, considered an important part of the disease, are rarely evaluated, partly for the same reason that makes it difficult to assess the progressivity of motor deficits.

Likewise, studying risk factors for PD in animal models can be challenging. As mentioned, understanding of the mechanisms underlying the influence of such factors on disease pathogenesis is crucial for the development of early diagnosis methods, allowing for preventive interventions. In addition, a similar response to known risk factors could add to the construct validity of animal models. Rodents do not present spontaneous PD. Thus, it is necessary to conduct studies that use a combination of variables that correspond to human risk factors with protocols that induce parkinsonian-like conditions in rodents.

**Figure 1:**
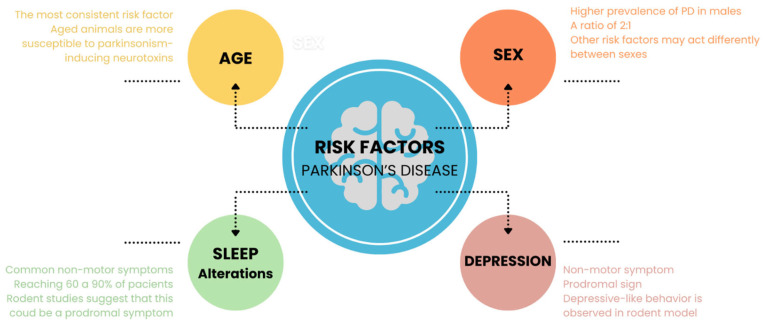
The study of risk factors and/or prodromal signs related to PD in animal models: advanced age, male sex, sleep alterations, and depression.

Overall, literature reviews on animal models of Parkinson’s disease focus on the motor and neurodegenerative aspects of the condition. The present narrative review aims to draw attention to the importance of studying risk factors and prodromal signs of PD in animal models. To this purpose, animal studies that investigated four of the main risk factors and/or prodromal signs related to PD were discussed: advanced age, male sex, sleep alterations, and depression. An overview of the content discussed here is schematized in Figure 1.

## 2. Methods

This review discusses articles retrieved from Embase, Google Scholar, Medline, and Pubmed until December 2023, coupled with an examination of citations from relevant articles. Main terms (“Parkinson’s disease AND risk factors” OR “Parkinson’s disease AND animal model” OR “movement disorder AND risk factors AND sex, age, depression, sleep disturbances”) were used to search for relevant articles that investigated four risk factors related to PD (age, sex, sleep alterations, and depression). Furthermore, to perform a broad search, synonyms and truncated terms of the descriptors were added to the search strategy. The search was restricted to English-language articles. The title and abstract of the studies were analyzed separately by three authors, who excluded articles unrelated to the topic. Moreover, the selected studies were further revised through full-text screening. The final reference list was generated based on relevance to the topics covered in this review.

## 3. Parkinson’s Disease

Parkinson’s disease (PD) is the most common neurodegenerative motor dysfunction and the second most prevalent neurodegenerative disease associated with aging [8,9,10,11], and it has a higher incidence in men than in women [9]. PD is a multifactorial disease with no defined etiology. The underlying pathophysiology is characterized by the progressive death of dopaminergic neurons, leading to motor disorders such as bradykinesia, tremor at rest, muscle rigidity, and changes in posture and gait as the disease progresses [12,13,14]. However, studies show that in the early stages of the disease, PD patients present several non-motor dysfunctions, such as cognitive deficit, sleep disturbance, anxiety, depression, hyposmia, and constipation, which are related to other neuronal mechanisms such as noradrenergic and serotoninergic pathways [15,16,17,18].

There is a body of evidence suggesting that the factors involved in the pathogenesis of this disease interact to culminate in the neurodegenerative process. In other words, PD is defined as a condition of multifactorial etiology [19,20,21]. Currently, the diagnosis of PD relies on well-defined clinical criteria based on the cardinal motor symptoms that characterize the disease, and there are no standardized biomarkers for a possible early diagnosis [19,22]. Because of this, it is estimated that the diagnosis of PD is made several years after the beginning of the neurodegeneration process [23]. Thus, a possible PD diagnosis before the characteristic motor manifestation is of great relevance. Alternatively, the identification of individuals who are at high risk for this disease would pave the way for potentially effective preventive strategies in controlling or delaying neurodegeneration. For this purpose, it is important to characterize initial symptoms (usually non-motor, which may occur years before conventional diagnosis) and individual and environmental risk factors, as well as the interaction between risk factors and pathophysiological mechanisms of the disease [24,25,26,27].

## 4. Risk Factors for Parkinson’s Disease

Risk factors for PD are mainly studied through human epidemiological surveys [21]. Among all the conditions positively associated with the risk of PD, some are classic risk factors and others have pathogenic relevance, that is, they are considered initial symptoms of the disease. Risk factors are characteristics of the individual or the environment that increase the likelihood of the presence of the disease. Features with pathogenic relevance are the so-called prodromal markers. A priori, those markers are part of the pathological condition, such as non-motor signs that precede motor symptoms [27]. However, the identification of a risk factor or prodromal sign of PD is sometimes hampered by the absence of a method to identify the onset of the disease before the appearance of motor symptoms [19].

Regardless of their straight category, all these characteristics are highly relevant for identifying individuals at high risk [19,27]. As mentioned, the non-motor symptoms that precede motor impairment are considered prodromal signs that may result from the initial neurodegenerative process. Nevertheless, there is a possibility that these signs are pre-morbid, that is, occurring prior to the actual onset of the disease. In that case, they would qualify as risk factors [28,29]. Importantly, many of these signs are not routinely checked, which often leads to underdiagnosis [19]. In addition, these symptoms may be present in other pathological conditions or even in normal aging, unrelated to PD [30]. Finally, the pathophysiological mechanism underlying these symptoms is still unknown, and the time course of their emergence can vary between individuals and different populations at risk [25,27,31].

In epidemiological studies, evidence does not allow for a precise assessment of the chronology and causality of these events. In fact, except for age, no risk factor for PD has indisputable evidence of causality [21]. Furthermore, although many studies propose to survey such factors, little is known about the nature of their interaction with the mechanisms that initiate neurodegeneration [27]. In addition, one difficulty of epidemiological approaches is that several studies are based on self-reports of PD history instead of standardized clinical diagnoses [32,33,34,35,36,37,38,39,40]. Patients with other neurological diagnoses may present parkinsonism as a symptom, and even if the diagnosis is correct, there are subtypes of parkinsonism that can have different etiologies [41].

Other limitations regarding the precision of epidemiologic approaches to studying risk factors are: (1) geographical differences in the prevalence of the disease, which makes it difficult to compile studies carried out in different continents or countries [42]; (2) variability in the duration of follow-up for the study population [19]; and (3) heterogeneity in the clinical manifestation of PD [19]. In a very comprehensive review of meta-analyses, Bellou et al. [20] concluded that there is a large body of evidence in favor of associating PD risk with the most studied risk factors in the literature. However, in most cases, they cannot exclude possible methodological biases or alternative explanations. In particular, inverse causality has been pointed out as a possible reason for some risk factors. For example, the still poorly understood pre-motor phase of the disease could influence the patient’s habits or personality, resulting in exposure to certain environmental factors [43].

Finally, the relevance of studying risk factors lies not only in the possibility of preventive interventions but also in contributions to the investigation of pathophysiological mechanisms and the identification of therapeutic targets. Considering the above, it is essential to characterize risk factors for PD in animal models. Such studies could enable the investigation of the causality of risk factors, specify their temporal course, determine their prodromal role, and contribute to the unravel of the biological mechanisms responsible for the increased risk.

A large number of risk factors have already been reported for the development of PD [44]. Among the risk factors in epidemiological studies, the most significant is undoubtedly advanced age. In addition, the male sex, family history, the use of pesticides/herbicides and associated activities, sleep changes, and depression can be highlighted. Additionally, studies point to other factors such as ethnicity, high consumption of dairy products, and traumatic brain injury.

In this review, two important risk factors mentioned in the literature will not be addressed: family history and the use of pesticides. There is an influence of genetic inheritance on the development of PD [45], which results in a positive association between family history and the risk of developing the disease [19,46]. Cases of monogenic inheritance determining PD occur in only about 5% of patients. Therefore, the expressive association between family history and the risk of PD is probably due to the inheritance of multiple susceptibility loci that predispose to the development of the disease [19,21,41,45,47]. However, the natural aspect of this influence cannot be studied in rodent models because these species do not develop PD spontaneously. Nevertheless, studies on genetically modified animals have been carried out to model PD [48,49].

The use of pesticides and related activities (rural life, agricultural work, etc.) have been repeatedly identified as factors that increase the risk of developing PD [19,21,44,50,51,52], but not always [21,41,53]. Some compounds have been used in animal studies to induce PD, such as rotenone and paraquat [54,55,56]. These approaches have some advantages over other animal models, such as the presence of alpha–synuclein aggregates [57]. However, there are some constraints in studying them as risk factors in laboratory animals: (1) the extremely high toxicity of these compounds, which compromises the survival rates of the animals under study [58] and (2) the fact that pesticides have been reported as causal factors of parkinsonian symptoms in humans [59,60]. This causal relation would characterize this condition as drug-induced parkinsonism, not exposure to a risk factor for idiopathic PD.

Thus, this study will focus on: (1) age and male sex, well-known risk factors for PD, and (2) sleep disturbances and depression, conditions that have been proposed both as risk factors and prodromic characteristics of the disease [19,61,62,63]. First, main pharmacological models for PD will be briefly described. Then, the state-of-the-art of each of the risk factors in the context of animal studies will be discussed. The main results regarding such risk factors obtained in pharmacological rodent models—and discussed in the present review—are summarized in Table 2.

## 5. Neurotoxic and Pharmacological Rodent Models for PD

Currently, two types of animal models are most used to study PD: genetic models, based on the expression of genes related to the disease; and neurotoxic/pharmacological models, which use drugs that interfere with dopaminergic transmission. Among the toxins most used in animal models of PD, 6-hydroxydopamine (6-OHDA) stands out. This toxin enters dopaminergic and noradrenergic neurons through dopamine and noradrenaline transporters, respectively. However, it is worth mentioning that 6-OHDA does not cross the blood–brain barrier. Therefore, it is necessary to administer this substance directly to the animal’s central nervous system. Once inside dopaminergic neurons, the toxin acts mainly on the mitochondrial complex I of the respiratory chain, leading to oxidative stress and degeneration of neurons in the substantia nigra pars compacta (SNpc) and ventral tegmental area (VTA) [76,77,78].

Another toxin used to induce PD is 1-methyl-4-phenyl-1,2,3,6-tetrahydropyridine (MPTP), the precursor of 1-methyl-4-phenylpyridine (MPP+), a neurotoxin capable of causing damage in the nigrostriatal pathway. MPTP crosses the blood–brain barrier and is subsequently converted to MPP+ by monoaminoxidase-B (MAO-B), leading to the blockage of the electron transport chain in the mitochondrial complex I of the respiratory chain, which results in oxidative stress [76,78,79].

Rotenone is a neurotoxin used as a pesticide that can also be used as a PD inducer in animals. This toxin crosses the blood–brain barrier and, similar to the other already mentioned toxins, acts on the mitochondrial complex I of the respiratory chain [80], in addition to preventing cell proliferation and blocking the mitosis process [81]. Chronic administration of this substance in rats induces nigrostriatal neurodegeneration, as well as the formation of Lewy bodies [82].

Reserpine is an alkaloid extracted from *Rauwolfia serpentina*, used in the first animal model of PD [83]. Since then, this model has been widely used to test new treatments to PD. Reserpine blocks the vesicular membrane transporters responsible for the storage of monoamines (dopamine, norepinephrine, and serotonin) in the synaptic vesicles, thus causing the depletion of these neurotransmitters [84]. One of the limitations of this model is that reserpine not only depletes dopamine but also other monoamines. However, this animal model can mimic the biochemical and behavioral effects of the disease, since the reduction of striatal dopamine promotes symptoms such as akinesia, tremors, and cognitive deficits [85]. In addition, there is evidence that PD involves the impairment of multiple neurotransmission systems, including serotonin [17,86] and norepinephrine; [87,88]. Therefore, the involvement of neurotransmitters other than dopamine in the reserpine model of PD seems to be relevant for the construct validity of this model.

**Figure 2:**
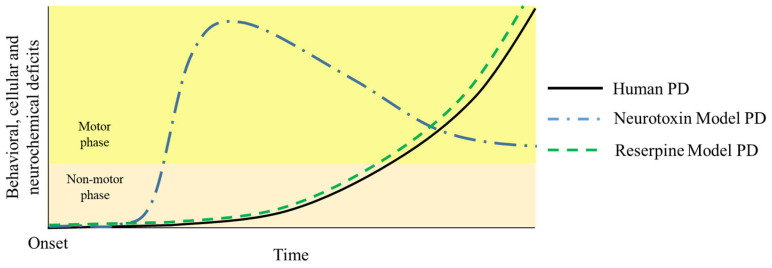
Schematic comparison of the progression of behavioral, cellular, and neurochemical deficits in the non-motor and motor phases of human PD and in pharmacological rodent models.

Originally, reserpine doses commonly used to induce parkinsonism in animals ranged from 1 to 10 mg/kg [89,90,91]. Such doses induce intense and immediate motor impairment. In the early 2010s, the repeated administration of a lower dose (0.1 mg/kg) was proposed. This protocol showed a gradual impairment in catalepsy in the bar test, in traveled distance in the open field, and orofacial movements occurring progressively over treatment [2,92,93]. Furthermore, Santos et al. [92] also demonstrated that the motor alterations presented by the animals were preceded by cognitive disabilities. In addition, a reduction in the tyrosine hydroxylase (TH) labeling and an increase in lipid peroxidation and neuroinflammatory parameters in the nigrostriatal dopaminergic pathway were also demonstrated [92,93]. A comparison between the progression of parkinsonian alterations induced by acute neurotoxins versus chronic reserpine is illustrated in Figure 2.

## 6. Age

Age has been linked to PD since the original description by James Parkinson. Currently, it is considered the most consistent risk factor for the disease [27,43,94,95]. A meta-analysis study reported that the prevalence ranges from 41 individuals per 100,000 in the 40 to 49 age group to 1900 individuals per 100,000 for the population over 80 [96]. Kim et al. [96] observed a prevalence of 1229 individuals per 100,000 for the Latin American population over 60. Indeed, advanced age is the most applicable factor for the inclusion of an individual in the population at risk for PD, along with family history [30].

Although the association between neurodegenerative diseases and aging seems obvious, this link is not found for all diseases that fall into this category. For example, Huntington’s disease and amyotrophic lateral sclerosis occur in younger individuals. Thus, for those neurodegenerative diseases that are associated with aging (such as PD), there must be some intrinsic factor of the aging process that leads to (or accelerates) its onset. However, the mechanisms involved in the influence of age on the development of PD are not completely understood. It has been proposed that normal aging and the pathophysiology of PD have common cellular mechanisms. In other words, the cellular aging that normally occurs in the nigrostriatal dopaminergic pathway would be exacerbated in PD due to a combination of genetic and environmental factors [97]. However, this hypothesis is not supported by some studies, which argue that the processes of normal aging and degeneration underlying PD—and other age-related neurodegenerative conditions—occur through distinct mechanisms [98,99,100,101].

Although some researchers have addressed this issue in non-human primates [97] few rodent studies have approached the specific association between aging and PD, weakening the translatability of preclinical findings [102]. In the study by Gupta et al. [64], 21-month-old male C57BL/6 mice received two MPTP injections intraperitoneally, the first at a dose of 30 mg/kg and the second at 15 mg/kg (due to the death of many animals). Three-month-old young adult mice C57BL/6 also received two doses of MPTP (30 mg/kg). After euthanasia, the brain was sliced for immunofluorescence analysis. Histological analysis showed a marked reduction in fluorescence in noradrenergic neurons of the locus coeruleus and in dopaminergic neurons of SNpc and VTA of elderly mice when compared to young controls [64].

In another study, Tremblay et al. [65] showed that pre-treatment with cystamine—an antioxidant and anti-apoptotic molecule—two days prior, and during 14 days after MPTP lesioning, increased the immunostaining for tyrosine hydroxylase in the striatum, as well as Nurr1 gene expression and increased the density of dopamine transporter in the substantia nigra in aged rodents. Similarly, Patki et al. [66] demonstrated that elderly mice (6 to 10 months) showed deficits in the activity of the respiratory chain in mitochondria, decreased antioxidant enzymes and cytochrome c, and a significant reduction in TH and DA uptake transporter. In addition, the older animals had impaired movement when compared to younger mice (6–10 weeks) subjected to the same protocol. It is worth mentioning that these changes were detected up to six weeks after the chronic protocol.

It has been demonstrated that aged animals are more susceptible to MPTP, and the neurotoxin induces a more pronounced reduction of TH in the aged brain [67]. The same study reported that astaxanthin-treated aged mice, when exposed to the MPTP neurotoxin, exhibited a significant loss of tyrosine hydroxylase throughout the nigrostriatal circuit compared to young mice. This suggests that aged animals respond differently to the MPTP toxin due to greater vulnerability of the aging brain.

Using a progressive animal model of parkinsonism, based on the administration of repeated injections of a low dose of reserpine (0.1 mg/kg), Melo et al. [68] observed that elderly rats (18–24 months) were more susceptible to the effects of the treatment when compared to adult animals (6–8 months). Indeed, aged animals developed motor alterations earlier than adult animals. In addition to the more severe motor changes, the authors observed that elderly rats showed a reduction in TH immunoreactivity in SNpc, dorsal striatum, and VTA. Furthermore, after treatment interruption, older animals did not show reversibility of the behavioral and dopaminergic changes caused by reserpine, supporting the hypothesis that the use of older animals better represents the behavioral and pathophysiological changes observed in the progressiveness of PD.

## 7. Sex

The greater prevalence of PD in males is well-recognized and reported by numerous studies. The relative risk reaches a ratio of 2:1 [21,27,94,95,103,104]. Of relevance, this proportion varies according to the age group. For example, Taylor et al. [105] reported that the male/female sex ratio is higher in older age groups [105]. In addition to age, it is interesting to highlight that other risk factors may act differently between the sexes, such as coffee consumption, physical activity, and the use of non-steroidal anti-inflammatory drugs [21]. In addition to being less likely to develop the disease, women may have a more benign motor phenotype, with a slower progression compared to men [106]. Additionally, there is evidence that the effectiveness of treatment with antiparkinsonian drugs depends on sex [107]. On the other hand, some non-motor symptoms such as nociceptive alterations and depression seem to be more prominent in women [108], although these findings are controversial [109]. Thus, clarifying the mechanisms related to the differential susceptibility to PD between sexes may be relevant to improving possible preventive and therapeutic strategies.

Epidemiological studies show that the incidence of PD in men remains higher than in women, even with increasing age. The incidence rate in men over 40 years of age is 61.21 cases per 100,000 inhabitants, while in women of the same age group, the incidence is 37.55 cases. The incidence rate among women is constantly increasing, from 3.26 cases per 100,000 inhabitants up to 49 years old to 103.48 cases up to 80 years old, with the peak between 70 and 79 years old. In men, in the same age groups, the incidence rate increases from 3.37 to 258.47 cases per 100,000 inhabitants, respectively, and this rate increases as patient survival increases. However, different rates are shown when they are restricted to specific geographic regions [10,96,110,111]. In addition, women with PD experience difficulties in receiving treatment and caregiving [112].

Because the risk of developing PD is evidently lower in females, the hypothesis that estrogen would be a protective factor against the development of the disease was raised. Indeed, the neuroprotective action of this hormone has been reported [113,114]. On the other hand, epidemiological studies show that the association between estrogen levels and protection against the development of PD is controversial [21,43,115,116]. There is some evidence of an increased risk of PD in women who have undergone ovariectomy or hysterectomy, used oral contraceptives, or have abstained from hormone replacement therapy [104,117,118,119]. However, a very comprehensive meta-analysis and well-controlled prospective studies did not show significant associations between the risk of PD and the use of oral contraceptives, surgical menopause, or hormone replacement therapy [19,116]. As a possible cause of this controversy, it has been demonstrated that the neuroprotective effect of estrogen occurs in the preclinical phase, postponing the degenerative effects of the disease, but it does not have any effect once the symptoms are already established [106]. Nevertheless, if estrogen promoted neuroprotection, the male/female prevalence ratio would be higher only at earlier ages, since estrogen levels are higher in younger women. Thus, it is likely that other factors are involved in the higher prevalence in males, such as cultural issues or some type of genetic susceptibility linked to sex chromosomes, but these factors have not yet been specifically investigated [21].

The neuroprotective effects of female hormones, especially estradiol (17β-estradiol), in the pathophysiology of neurodegenerative diseases have been demonstrated in various animal models [120,121]. In addition, it has been shown that estradiol has anti-inflammatory properties [122]; prevents neuronal death by increasing the endogenous synthesis of anti-apoptotic molecules [123]; acts on mitochondria, improving bioenergetic activity and basal mitochondrial respiration [124,125]; and increases levels of brain-derived neurotrophic factor (BDNF), a key molecule involved in neuronal survival, neurotransmission, dendritic growth, and cell communication in the central nervous system. In astrocytes, estradiol has neurotrophic activity, facilitating the secretion of growth factors, represses the expression of glial fibrillary acidic protein (GFAP), and reduces astrogliosis [126,127]. Neuroprotective effects of progesterone have also been demonstrated in a variety of experimental models [128,129]. Progesterone attenuates blood–brain barrier dysfunction [130]; promotes the survival of newborn neurons [131]; has anti-inflammatory [132] and antioxidant [133] properties; acts in the preservation of mitochondrial functions [134,135]; reduces GFAP levels [136]; and regulates BDNF production and release [129]—which can be interpreted as neuroprotection mechanisms.

Studies on sexual differences in animal models of PD provide a more accurate control of hormonal variation since most human studies depend on personal reporting, and hormonal measurements are not carried out. In addition, other physiological factors that could be involved in the differences in prevalence between sexes can be investigated. Nevertheless, few animal studies have addressed this issue. For example, in the study by Field et al. [69], animals of both sexes presenting 6-OHDA-induced unilateral lesions were submitted to a test that evaluates vertical exploration in a confined cylinder. The results showed that 6-OHDA-treated male animals reduced the use of the hind limbs compared to females, despite the deficit in forelimbs movements being similar between sexes. In addition, males were more likely to contact the cylinder wall with their dorsal surface to keep an erect posture. The study also showed that female animals had a less severe reduction in the number of dopaminergic cells compared to males.

Using the repeated reserpine-induced progressive PD model, ref. [70] showed that females were more resistant to the deleterious effect of the treatment. Indeed, this sex did not present reduced TH immunoreactivity in the dorsal striatum and VTA. Also applying the reserpine-induced progressive protocol, ref. [71] showed that female animals did not present cognitive alterations and TH immunoreactivity reduction. In addition, females presented attenuated motor impairment compared to males. These findings reinforce the notion that more studies comparing sexes should be conducted to better comprehend the mechanisms that lead to neuroprotection in females.

## 8. Sleep Alterations

Sleep and circadian rhythm disturbances are among the most common non-motor symptoms in PD, reaching 60 to 90% of patients. For many years, this group of symptoms was considered only as secondary signs, unrelated to the pathophysiology of the disease, despite the high prevalence [137,138,139]. Even now, such disturbances are underreported or underrecognized by patients with PD [140]. A study showed that 20% to 30% of patients failed to report sleep disorders to their healthcare providers. The high rate of non-declaration of these disturbances by patients to healthcare providers means that many symptoms remain untreated. Factors that lead to help-seeking can be the acceptance of symptoms, lack of awareness that the symptom is associated with PD, and belief that no effective treatments are available [141].

More recently, attention to non-motor symptoms of PD has increased, especially to sleep and circadian rhythm disorders. Currently, these symptoms are recognized as important causes of quality-of-life impairment, and PD is known to affect important brain regions and neurotransmission systems related to the control of the sleep–wake cycle [142,143]. Sleep disorders present in DP are mainly insomnia, excessive daytime sleepiness (EDS), restless legs syndrome, circadian rhythm disorders, and rapid eye movement (REM) sleep behavior disorder (RBD) [144,145,146].

Insomnia is thought to be the most common sleep disorder in PD, with prevalence varying from 30% to 80%. Patients often report sleep fragmentation and early awakenings rather than sleep initiation difficulty. PD patients with insomnia are usually at more advanced stages of the disease, showing important motor, psychiatric, and autonomic symptoms [140]. Regarding RBD, studies estimate the prevalence at 23–25% in PD patients and 2–4% in the general population [140]. Another study shows a prevalence of RBD close to 50% in patients with PD. If REM sleep without muscle atony (preclinical form of the disorder) is also considered, the estimated prevalence is 60% in PD patients. Importantly, the highest predictive value for the future development of PD is observed for polysomnography confirmed RBD [147]. In addition, RBD is associated with a worse prognosis for patients. In other words, there is a higher risk of more severe motor dysfunction, hallucinations, cognitive impairment, and autonomic dysfunction [147].

Autopsy brain studies revealed the presence of Lewy bodies in the pedunculopontine tegmental nucleus (PPN), locus coeruleus/subcoeruleus complex, and gigantocellular reticular nucleus in the medulla oblongata of PD patients who previously developed idiopathic RBD [140]. These regions are considered part of the neural circuit that regulates atonia during REM sleep and are linked to RBD pathology [148].

Restless Legs Syndrome (RLS) is proposed as another important symptom of PD and is closely associated with periodic limb movement of sleep (PLMS). Studies evaluating the frequency of RLS and PLMS in PD patients generated very discrepant results, ranging from 0% to 52.3% of patients with the condition. Some studies showed that RLS is present in 14–16% of patients [96,149]. For some authors, RLS indicates an increased risk for PD. A 0.37% incidence of PD has been found in the RLS population, while in control individuals, the incidence of PD was 0.13% [140]. On the other hand, there are studies that found a similar prevalence of RLS among PD patients and in the general population [150]. These discrepant results can be related to the diagnosis accuracy. RLS diagnosis may be confounded in patients with PD due to the potential overlap of motor symptoms. Therefore, because the prevalence and causes of RLS in PD are still unclear, this disturbance has not been considered an accurate predictor.

Excessive daytime sleepiness (EDS) is present in 15–50% of PD patients. This condition is characterized by an urge to fall asleep during different daily-life circumstances, with a severe negative impact on the overall quality of life. The degeneration of hypothalamic orexin cells (related to vigilance maintenance) is an essential factor in PD-related EDS [120,151].

The mechanisms underlying the circadian fluctuation of symptoms are not known, although it is probably related to a circadian variation in central dopaminergic transmission [143]. The sleep regulatory centers and circadian rhythm circuits—such as the hypothalamus and various brainstem nuclei involved in sleep–wake regulation—are affected by the neurodegenerative process. Neuropathological changes in these regions may begin before the degeneration of the substantia nigra and may be related to many of the non-motor characteristics seen in PD, such as sleep and circadian rhythm disturbances [138,152].

Of relevance, many of these changes can occur prior to the appearance of motor symptoms and the diagnosis. Thus, the presence of such sleep disorders can classify an individual as at high risk for PD. The risk of developing PD is very high among patients who suffer from RBD [61], which is considered a prodromal sign of the disease [61,153]. RBD precedes the onset of parkinsonism by 13 years on average, but this interval can reach so as far as over 20 years [140,154]. However, the prevalence of this disorder in recently diagnosed patients is limited, and therefore, it is questioned whether RBD would be a pre-motor symptom in all cases of idiopathic PD [46,155]. Thus, the investigation of these sleep disturbances as risk factors becomes relevant. In addition, activities that interfere with sleep and circadian rhythm, such as night shift work, have also been suggested as risk factors for PD, although this is still under debate [156,157,158]. In this sense, it is not clear whether there is a causal relationship between sleep loss and an increased risk of PD, or if the pre-diagnosis period of the disease already has changes that would lead to sleep changes. Thus, the chronology of events remains to be clarified, and the study of the relationship between sleep deficits and PD in animal models could provide causal or mechanistic evidence. However, few studies using animal models seek to study the interaction between PD and sleep disorders.

Toxin-based animal models have greatly contributed to the development of symptomatic treatments, mainly for motor symptoms. Notwithstanding, some toxin-based models also show prodromal symptoms. For example, 6-OHDA and MPTP protocols, widely known for reproducing motor deficits accompanied by dopaminergic neuronal death, can mimic sleep disorders in animals. Increased muscle tone during REM sleep, which is suggestive of an RBD-like phenotype, was reported in rats treated with 6-OHDA and in rhesus monkeys and marmosets treated with MPTP [74].

It has also been shown that these pharmacological models can mimic the insomnia state present in patients with PD. Rats with a unilateral 6-OHDA lesion of the medial forebrain bundle show decreased sleep time during their inactive phase (light) of the 24 h light–dark cycle [73]. Increased wake time during the 12 h dark period has also been observed in rats with a selective 6-OHDA lesion of the SNpc [74]. Animals submitted to bilateral 6-OHDA lesion in the ventral tegmental area (VTA) show reduced REM sleep during the light period and an increase in total sleep time during the dark phase [72]. These findings are in line with some of the disturbances observed in PD patients, who are affected by insomnia at night and daytime sleepiness.

Genetic models based on changes in the α-syn gene (SNCA) have also been created in recent years. Many features of sporadic PD are observed in transgenic mice overexpressing wild-type α-syn. The model induces progressive changes in dopamine release and striatal content, alpha-synuclein pathology, and deficits in motor and nonmotor functions, including sleep disturbances [159,160]. It was recently demonstrated that A53T α-syn BAC transgenic mice present an RBD-like phenotype, hyposmia, and decreased TH-positive neurons in the SNpc. All of these findings were seen in the absence of motor deficits, suggesting that this could be a prodromal PD mouse model [160].

Sleep and circadian rhythm disorders may be a key component of the non-motor symptoms of Parkinson’s disease [161]. In addition, sleep and circadian rhythm disorders are difficult to reproduce in animal models of PD, although some studies have succeeded. On the other hand, one particularly interesting aspect of sleep disorders in PD is their potential impact on other symptoms, whether motor or non-motor alterations, and in quality of life [162], reinforcing the relevance of addressing the interaction between PD and sleep in animal studies.

## 9. Depression

Depression is present in approximately 40 to 60% of patients with PD, and the presence of this symptom worsens the already poor quality of life of individuals with this disease [163,164,165,166,167]. It is important to note that depression, or other mood disorders, can occur prior to the onset of motor symptoms [168], and that early treatment of depression associated with PD can promote better acceptance of the signs of the disease [169,170]. Despite that, depression in PD patients is still underdiagnosed and undertreated [167].

In addition to manifesting as a non-motor symptom in a large number of patients, a history of depression has a positive association with the subsequent development of PD [27,171,172,173]. Indeed, twice as much risk is reported for an individual with a history of depression to be later diagnosed with PD [19,174]. The presence of clinically diagnosed depression can be used as a criterion for including individuals in populations at risk in prospective studies [62].

The pathophysiology of depression associated with PD is not completely understood [164]. Some hypotheses intend to provide a pathophysiological explanation for the higher prevalence of depression in PD patients [175]. Considering depression as a prodromal sign, a hypothesis to explain its occurrence would be the degeneration of brainstem nuclei, midbrain, and cortex [29]. However, depression can also have other causes [176] and is positively associated with advanced age itself [177]. When depression occurs prior to the diagnosis of PD, it is unclear if it is part of the disease, or whether the individual with depression is more susceptible, and whether, together with other risk factors, this susceptibility would lead to the development of PD [175]. In epidemiological studies, in general, it is not possible to identify the chronology and causality of events, in the same way as discussed for symptoms related to sleep.

Another factor to be considered is that one of the main risk factors for depression is chronic stress [178]. In this respect, it has been suggested that chronic stress, by increasing nervous system susceptibility, could be a causal factor for neurodegeneration in PD [179,180]. Accordingly, there is evidence that stress is a risk factor for the development of PD [181,182,183].

The study of mechanisms related to the interaction between depression, stress, and the development of PD could contribute to refining the identification of individuals at risk and improving treatment strategies for patients who have depression as a relevant aspect during PD. However, this relationship has not yet been studied systematically in animal models. Although such studies could help clarify mechanisms underlying PD-related depression neuropathology, most of the animal models of the disease do not reproduce human disease progression and do not comply with nondopaminergic deficits [184].

In other words, the difficulty in approaching depression in animal models of PD relies on the same context of investigations involving non-motor symptoms. Indeed, those evaluations can be hindered by the presence of motor impairment. Nevertheless, an effort has been made to circumvent these difficulties.

One of the most used tests that addresses depression-like behavior in rodents is the forced swimming test. The animal is subjected to a container filled with enough water to necessitate swimming, without being able to support the hind paws at the bottom. The time the animals spend in immobility (i.e., not trying to escape the recipient by swimming) is considered a measure of learned helplessness and interpreted as depressive-like behavior. Almost all studies with neurotoxin models of Parkinson’s disease showed a decreased swimming time and/or increased immobility time (see [185] for review). However, in those cases, it is not possible to separate the motor deficit from the effects on depression per se.

Anhedonia is a well-known depression sign [186] that can be addressed in rodents by the sucrose preference test [187]. A choice between regular water and sucrose solution is offered to rats or mice, which regularly prefer sucrose. If the preference is not observed, this is interpreted as anhedonic behavior, and hence considered depressive-like behavior. This behavior would be less affected by motor impairment (at least one that is not too severe). If only the sucrose preference, and not the total amount of drink, changes with neurotoxin treatment, the effect is probably specific for depressive-like behavior and not motor function. Studies with neurotoxic PD models have shown a decrease in rodent sucrose preference compared to controls, but this effect was not unequivocal, as some studies also showed no changes in sucrose preference [188]. This study will not provide details of previous work as a very comprehensive review on studies that address depression in animal models of PD has already been done [188]. Nevertheless, progressive PD models could be a more interesting approach to investigate depression because it could be assessed in different stages of the progression, including those with no or little motor deficit. For example, Soares et al. [75] investigated the relationship between the predisposition to depressive-like behavior and the development of motor alterations in the progressive model of PD in mice induced by reserpine. Animals were classified into groups of depressive-like profiles and received a low dose (0.1 mg/kg) of reserpine over 40 days. Anhedonic behavior was considered a depressive-like trait, and each mouse was submitted to the sucrose preference test. Based on their performance, mice were allocated into three groups: those with greater depressive-like behavior (predisposed), those with less depressive depressive-like behavior (non-predisposed), and those with intermediate levels. Only animals categorized at the extremes of the depressive-like spectrum were further divided into two subgroups, reserpine-treated or vehicle-treated. The catalepsy and oral movement tests were used to assess motor alterations, while the open field test was used to evaluate exploratory activity. Reserpine induced parkinsonian motor deficits. However, there were no differences between animals with different depressive-like behavior profiles. Thus, it was not possible to establish a relationship between parkinsonism and the propensity for depression based on the basal sucrose preference test under those experimental conditions. Thus, although depressive-like behavior is seen in animals that went through parkinsonism induction, more studies are needed to verify if a depressive profile could predispose the animals or increase susceptibility to alterations induced by PD models.

## 10. Conclusions

The increasing incidence of PD, combined with a lack of specific knowledge on risk factors, impacts a substantial number of individuals worldwide. There is a recent effort in finding biomarkers that could provide pre-symptomatic diagnosis of PD, including single-photon emission computed tomography imaging, positron emission tomography, olfactory alterations, skin and colonic biopsy, changed metabolites, gene sequencing, and α-synuclein quantification in body fluids [188,189,190,191,192,193]. Nevertheless, the identification of risk factors and evaluation of prodromal signs, together with family history, are still the main methods to classify individuals at risk for this disease. Human epidemiological studies are useful in this search, but this approach fails in providing causality.

The aim of the present review was to provide an overview of experimental animal studies related to the four main risk factors of PD—age, sex, sleep alterations, and depression. Overall, the review summarizes the available evidence, pointing to the need for a greater number of animal studies focusing on PD risk factors. Importantly, although several animal models have helped clarify PD pathophysiology, up to date, none of them has completely reproduced the entire natural history of the disease. An ideal model of prodromal PD would be one that reproduces various PD-specific premotor symptoms followed by the slowly progressive DA neurodegeneration. In conclusion, studies that aim to investigate well-known risk factors for PD in animal models can help elucidate mechanisms related to the disease’s etiology and contribute to future prevention or treatment approaches. Therefore, continuing to study risk factors and prodromal signs in animal models of PD is crucial.

## 11. Limitations of the Study

It is important to emphasize that this review does not intend to close the issue of studying risk factors for PD in animal models. Here, only four of the main risk factors were addressed, and others must be considered when approaching the subject. Furthermore, this work did not undertake a systematic approach. Therefore, more attention needs to be paid to factors such as variations in animal model used, length of protocols, species, and treatment in the studies carried out for each risk factor. Greater reliability in literature findings regarding how risk factors influence the onset and progression of PD will be beneficial for the development of new prevention and treatment approaches.

## Figures and Tables

**Table 1:** Quantitative summary of pre-clinical studies on Parkinson’s disease carried out in animal or cell culture models *.

Model	First Publication (Year)	Number of Published Studies	Percentage (%)
Reserpine	1950	530	2.58
Haloperidol	1962	963	4.69
6-OHDA	1975	5242	25.50
Genetic	1977	2869	13.96
Cell culture	1979	2367	11.52
MPTP	1983	6735	32.77
Rotenone	1987	1847	8.99

* Search and selection by the title of the articles until December 2023. Pubmed database. Terms of search: “drug name” and “rat” or “mice” (rodent models); “cell culture”; and “Parkinson”.

**Table 2:** Risk factors and main results obtained in cited articles of pharmacological rodent models for PD.

Investigated Factor	Strain	Pharmacological Model	Measures	Outcomes	Publication
Age	Male mice C57BL/6	MPTP injection in elderly and young animals	Histological	Reduction in fluorescence in noradrenergic neurons of the locus coerulus and dopaminergic neurons of SNpc and VTA.	Gupta et al., 1986 [64]
	Male mice C57BL/6	MPTP injection in elderly animals	Histological	Reduction of immunostaining for tyrosine hydroxylase in the striatum, as well as Nurr1 gene expression, and increased density of dopamine transporter in SN.	Tremblay et al., 2006 [65]
	Male mice C57BL/6	MPTP injection in elderly and young animals	The mitochondrial content of ATP; Histological; and Behavioral test	Deficits in the activity of the respiratory chain in mitochondria, decreased antioxidant enzymes and cytochrome c, and a significant reduction in TH and DA uptake transporter. In addition, the older animals had impaired movement when compared to younger mice.	Patki et al., 2009 [66]
	Male mice C57BL/6	MPTP injection in elderly and young animals	Histological	Loss of tyrosine hydroxylase throughout the nigro-striatal circuit compared to young mice.	Grimmig et al., 2018 [67]
	Male Wistar rats	Repeated injections of a low dose of reserpine	Histological and Behavioral tests	Elderly animals were more susceptible to the effects of the treatment compared to adult animals. Elderly rats developed motor deficits earlier than adult rats. Elderly rats showed a reduction in tyrosine hydroxylase immunoreactivity in SNpc, striatum, and VTA.	Melo et al., 2022 [68]
Sex	Female and Male Long-Evans rats	6-OHDA injection	Histological and Behavioral tests	Male animals reduced the use of their hind limbs compared to females, despite the deficit in forelimb movements being similar between sexes. In addition, males were more likely to contact the cylinder wall with their dorsal surface to keep an erect posture. Female animals had a less severe reduction in the number of dopaminergic cells compared to males.	Field et al., 2006 [69]
	Female and Male Wistar rats	Repeated injections of a low dose of reserpine	Histological and Behavioral tests	Females were more resistant to the deleterious effects of the treatment. Indeed, this sex did not present reduced TH immunoreactivity in the dorsal striatum and VTA.	Bispo et al., 2019 [70]
	Female and Male Wistar rats	Repeated injections of a low dose of reserpine	Histological and Behavioral tests	Female animals did not present cognitive alterations and TH immunoreactivity reduction. In addition, females presented attenuated motor impairment compared to males.	Lima et al., 2021 [71]
Sleep	Male Wistar rats	6-OHDA injection	Polysomnographic recordings	Rats with bilateral 6-OHDA lesion in the VTA show reduced REM sleep during the light period and an increase in total sleep time during the dark phase.	Sakata et al., 2002 [72]
	Male Sprague-Dawley rats	6-OHDA injection	Polysomnographic and video recordings	Rats with a unilateral 6-OHDA lesion of the medial forebrain bundle show decreased sleep time during their inactive phase (light) of the 24 h light–dark cycle.	Vo et al., 2014 [73]
	Male Sprague-Dawley rats	6-OHDA injection	Polysomnographic recordings	Rats with bilateral 6-OHDA lesion in the caudoputamen increased wake time during the 12 h dark cycle. These animals exhibited sleep–wake fragmentation and reduced diurnal variability of sleep.	Qiu et al., 2016 [74]
Depression	Mael Swiss mice	Repeated injections of a low dose of reserpine	Behavioral test	No differences were observed between animals with different depressive-like behavior profiles.	Soares et al., 2021 [75]

## Data Availability

Not applicable.
